# GSK3β inhibition attenuates LPS-induced IL-6 expression in porcine adipocytes

**DOI:** 10.1038/s41598-018-34186-0

**Published:** 2018-10-29

**Authors:** Linjie Wang, Xueying Li, Yan Wang

**Affiliations:** 1 College of Animal Science and Technology, Sichuan Agricultural University, Chengdu, Sichuan P.R. China; 2 Farm Animal Genetic Resources Exploration and Innovation Key Laboratory of Sichuan Province, Sichuan Agricultural University, Chengdu, Sichuan P.R. China

## Abstract

IL-6 is not only a proinflammatory cytokine associated with inflammatory responses but also a regulator on the energy and glucose metabolism in adipose tissue. Glycogen synthase kinase 3β (GSK3β) has fundamental roles in the regulation of pro- and anti-inflammatory cytokines production. However, the regulatory role for GSK3β in the pig inflammatory response in adipocytes remains unknown. We show here that SB216763 and LPS increased the phosphorylation of GSK3β (Ser9), and decreased the phosphorylation of GS (Ser641) in adipocytes. The activity of porcine GSK3β was inhibited by SB216763, an inhibitor of GSK3β, attenuated the production of IL-6 in LPS-stimulated adipocytes. Additionally, the essential core region of the pig IL-6 promoter located at −191 bp to −59 bp, and an NF-κBp65 element in this region was responsible for IL-6 promoter activity. The transcription activity of NF-κBp65 was activated by LPS stimulation, and the GSK3β inhibition repressed LPS-induced luciferase activity of the IL-6 promoter. Furthermore, LPS increased p65 binding to the NF-κB site, and GSK3β inhibition had no effect on the association of NF-κBp65 with IL-6 gene promoter after LPS treatment. These results demonstrate that GSK3β has important regulatory roles in the LPS-induced inflammatory response of IL-6 production in pig adipocytes.

## Introduction

Interleukin-6 (IL-6) is originally identified as a B-cell stimulatory factor^1^ and has important functions in regulating the immune response, hemopoiesis and inflammation^2^. IL-6 is a pro-inflammation cytokine mainly produced by various types of cell including stimulated monocytes, macrophages, T cells and epithelial cells^3^. Glycogen synthase kinase 3 (GSK3) is serine/threonine kinase, and identified as a regulator in the innate and adaptive immune system^4^. The phosphorylation of GSK3α (serine21) and GSK3β (serine9) has been reported to affect the activity of GSK3 in immune cells^5^. GSK3 activity is inhibition by phosphorylation of Ser21 in GSK3α or Ser9 in GSK3β. The crucial role of GSK3β in inflammation is established by the finding that active GSK3β is necessary for pro-inflammatory cytokine production following TLR stimulation^6^. The inhibition of GSK3β by LiCl significantly induces the production of IL-10 and IL-12 compared with the untreated condition, but this induction is significantly elicited by LPS stimulation in PK-15 cells^7^.

In normal immune cells, GSK3β does not affect the production of inflammatory cytokines. In contrast, in LPS-stimulated human monocytes, the inhibition of GSK3β increases the production of anti-inflammatory cytokines and reduces the expression of pro-inflammatory cytokines^6,8^. In Mycobacterium bovis BCG, it is demonstrated that GSK3β inhibition increases the production of IL-10 through the PI3K-Akt signaling in primary human blood monocytes (PHBM)^9^. In LPS-induced glia, GSK3 mediates inflammatory cytokine levels in the culture medium, with the activity change of the GSK3β isoform, and demonstrates a vital role of GSK3β as a modulator of inflammatory cytokine levels in the brain^10^. In an air pouch GAS infection mouse model, the administration of GSK3β inhibitor significantly reduces the level of serum TNF-α and improved the survival rate^11^. These findings indicate a significant role for GSK3β in the inflammatory response caused by bacterial pathogen via inflammatory cytokines expression. However, the roles for GSK3β in the inflammatory response in adipocytes have not yet fully investigated.

In the pig, two GSK3 isoforms (GSK3α and GSK3β) have been isolated from liver tissues^12,13^. Previous studies have shown that five GSK3β isoforms are identified in pig different tissues and were differentially regulated during the course of the insulin treatment in PK-15 cells^14^. GSK3β regulates expression of pig GYS1 gene through NF-κBp65, and overexpression of GSK3β reduces the association of NF-κBp65 with GYS1 gene promoter^15^. However, the regulatory role for GSK3β in the pig inflammatory response in adipocytes remains unknown. The main purpose of this study was to investigate the regulatory role of GSK3β on LPS-induced IL-6 production in the pig adipocytes. In this study, LPS inhibited the activity of GSK3β, increasing the IL-6 production. The transcription activity of NF-κBp65 was activated by LPS stimulation, and the GSK3β inhibition repressed LPS-induced luciferase activity of the pig IL-6 promoter. The results of this study provide an insight into understanding the functions of GSK3β in the LPS-induced inflammatory response of IL-6 production in pig adipocytes.

## Results

### SB216763 and LPS increased the phosphorylation of GSK3β (Ser9) and decreased levels of phosphorylation of GS (Ser641)

**Figure 1:**
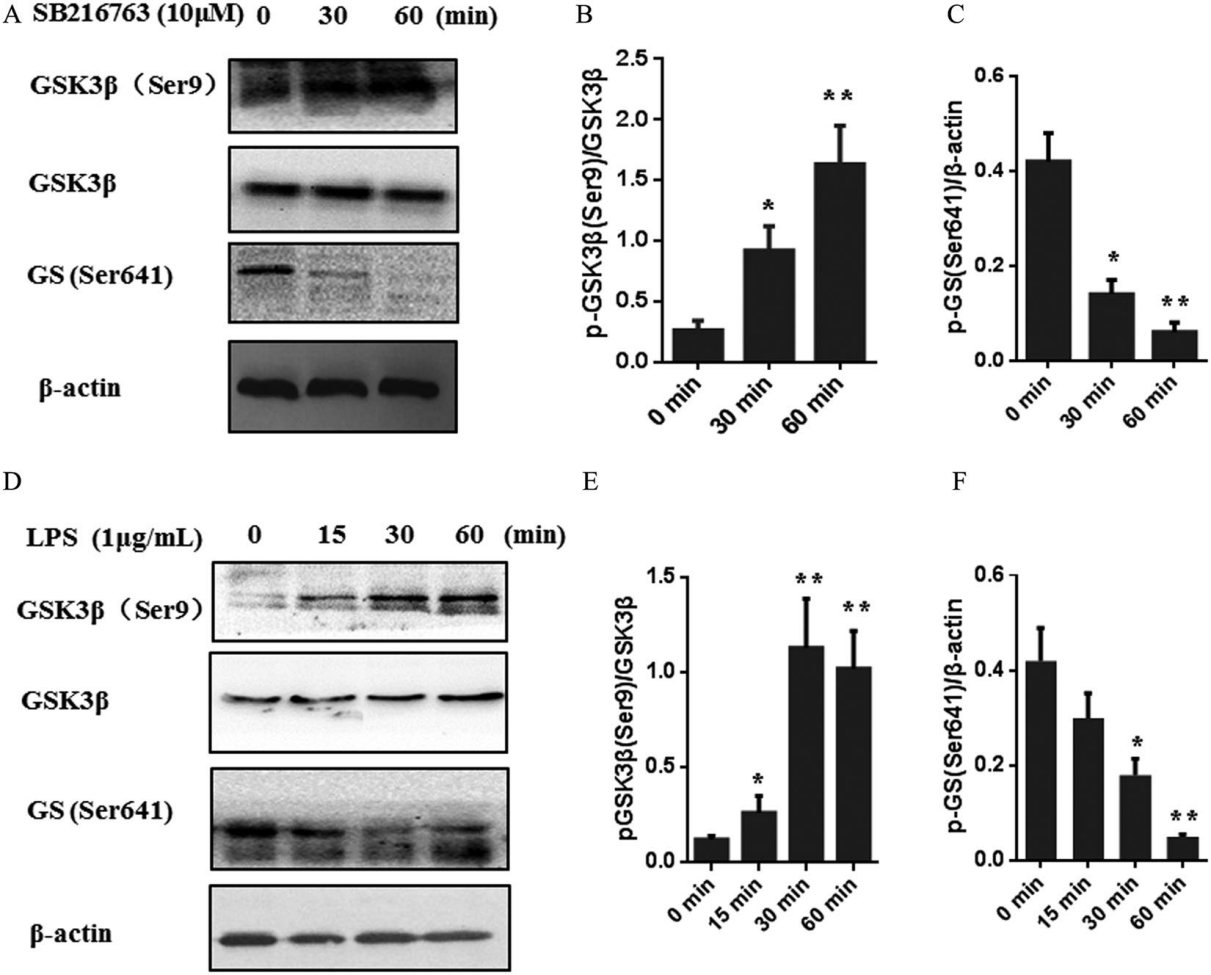
Phosphorylation of GSK3β (Ser9) and dephosphorylation of GS (Ser641) was induced by SB216763 and LPS in pig adipocytes. (**A**–**C**) Representative western blots and quantified results of the phosphorylation levels of GSK3β (Ser9) and GS (Ser641) in pig adipocytes treated with GSK3β inhibitor, SB216763 (10 μM) for 0, 30, 60 min. (**D**–**F**) Representative western blots and quantified results of the phosphorylation levels of GSK3β (Ser9) and GS (Ser641) in pig adipocytes treated with LPS (1 μg/mL) for 0, 15, 30, 60 min. β-actin was used as the loading controls. Data Bar graphs are expressed as means + SEM of phosphorylation levels of GSK3β (Ser9) normalized to total GSK3β and GS (Ser641) normalized to β-actin from three independent cultures.

To determine the effect of SB216763 and LPS on GSK3β activity, we assessed the phosphorylation of GSK3β (Ser9) and GS (Ser641). Previous studies showed that the activity of GSK3β is negatively regulated by phosphorylation of serine residues 9 (Ser9)^16^, and glycogen synthesis (GS) is recognized as a direct substrate of GSK3β and the activity regulation of GS is to dephosphorylate it^17^. Firstly, we determined the effectiveness of SB216763 on GSK3β. As shown in Fig. 1A,B, the phosphorylation of GSK3β (Ser9) was significantly (*P* < 0.01) up-regulated after SB216763 treatment, reaching a peak at 60 min. Total GSK3β did not change in response to SB216763 treatment. In contrast, glycogen synthase (GS) exhibited the highest phosphorylation (Ser641) level at 0 min, and a significantly (*P* < 0.01) decrease in phosphorylation levels of GS (Ser641) was observed at 60 min (Fig. 1C). The results demonstrated that SB216763 increased levels of phosphorylation of GSK3β (Ser9) and then inhibited the kinase activity of GSK3β.

We next assessed how LPS-mediated the phosphorylation of GSK3β (Ser9) and GS (Ser641). As shown in Fig. 1D, the phosphorylation of GSK3β (Ser9) and GS (Ser641) changed in a time-dependent manner during the course of the LPS treatment. The phosphorylation of GSK3β (Ser9) was significantly (*P* < 0.01) up-regulated after LPS induction, reaching a peak at 60 min (Fig. 1E). In contrast, the phosphorylation of GS (Ser641) was significantly (*P* < 0.01) down-regulated after induction, attaining the lowest level at 60 min (Fig. 1F). Total GSK3β did not change in response to LPS treatment. The results demonstrated that LPS increased levels of phosphorylation of GSK3β (Ser9) and decreased levels of phosphorylation of GS (Ser641), and then inhibited the kinase activity of GSK3β.

### The inactivation of GSK3β decreased the supernatant IL-6 productions in LPS-simulated adipocytes

**Figure 2:**
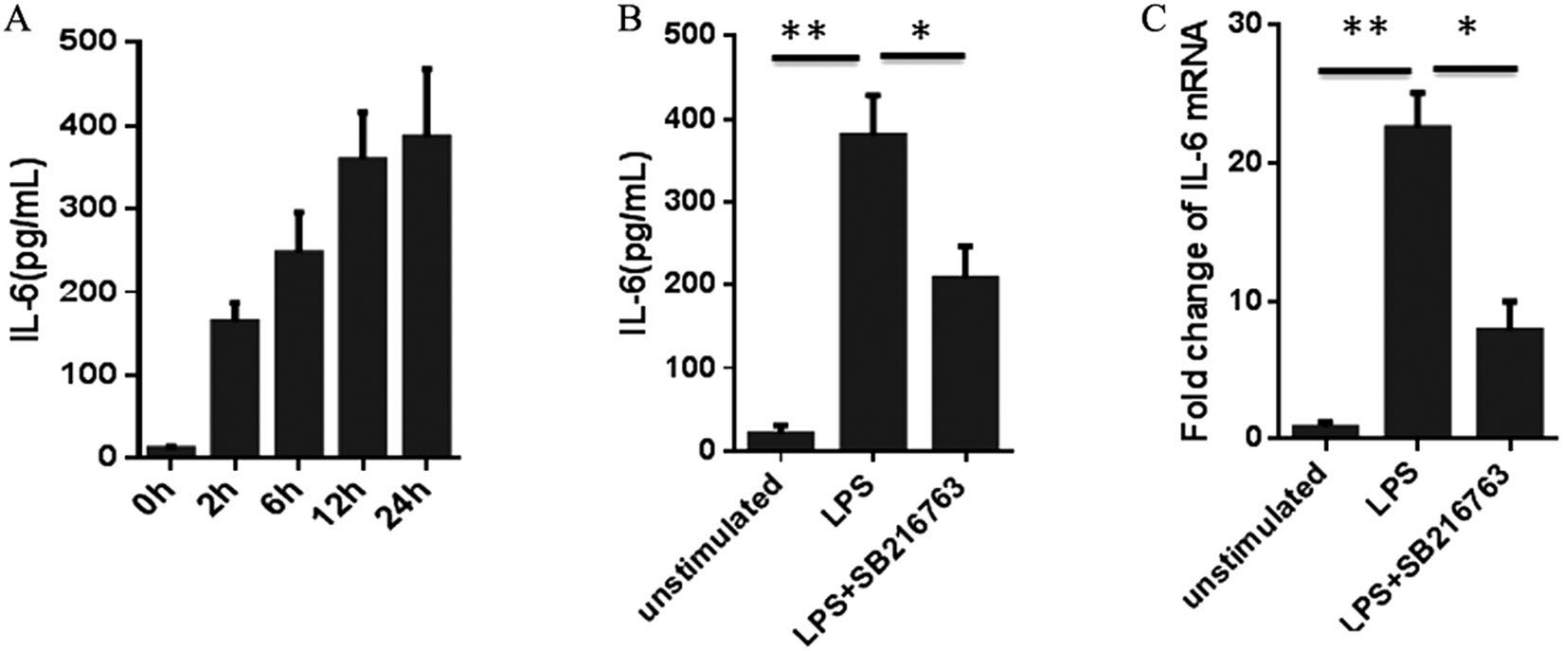
Effect of GSK3β inhibition on LPS-induced IL-6 production in adipocytes. (**A**) Adipocytes were pretreated with SB216763 for 60 min, then left unstimulated or stimulated with 1 µg/ml of LPS for 24 h. IL-6 in cell-free supernatants was analyzed by ELISA. (**B**) Total RNA was extracted and mRNA expression levels of IL-6 were measured by qPCR. Data are presented as the means ± SEM.

To investigate the inflammation role that GSK3β acted in LPS-stimulated adipocytes, we detected the culture supernatant productions of IL-6 via ELISA. As shown in Fig. 2A, the production of IL-6 were very low at 0 h, then LPS markedly increases the amount of IL-6 in culture supernatants, reaching its highest level at 24 h. The results indicated that LPS significantly (*P* < 0.01) induced the IL-6 expression amount in culture supernatants. In addition, we used the inhibitor of GSK3β by SB216763 to further confirm the role that GSK3β acted in LPS-stimulated inflammation response in adipocytes. SB216763 attenuated the production of IL-6 (*P* < 0.01) induced by LPS (Fig. 2B).

We next determined whether GSK3β regulated the mRNA expression level of IL-6 in LPS stimulated mature adipocytes. The results showed that LPS significantly (*P* < 0.01) increased the expression of IL-6. Additionally, the inhibition of GSK3β abolished (*P* < 0.01) the ability of LPS to induce the mRNA expression level of pig IL-6 gene (Fig. 2C). These results demonstrate that the inhibition of GSK3β activity decreased the IL-6 production by LPS-stimulated adipocytes.

### Identification of crucial transcriptional factors controlling IL-6 gene expression

**Figure 3:**
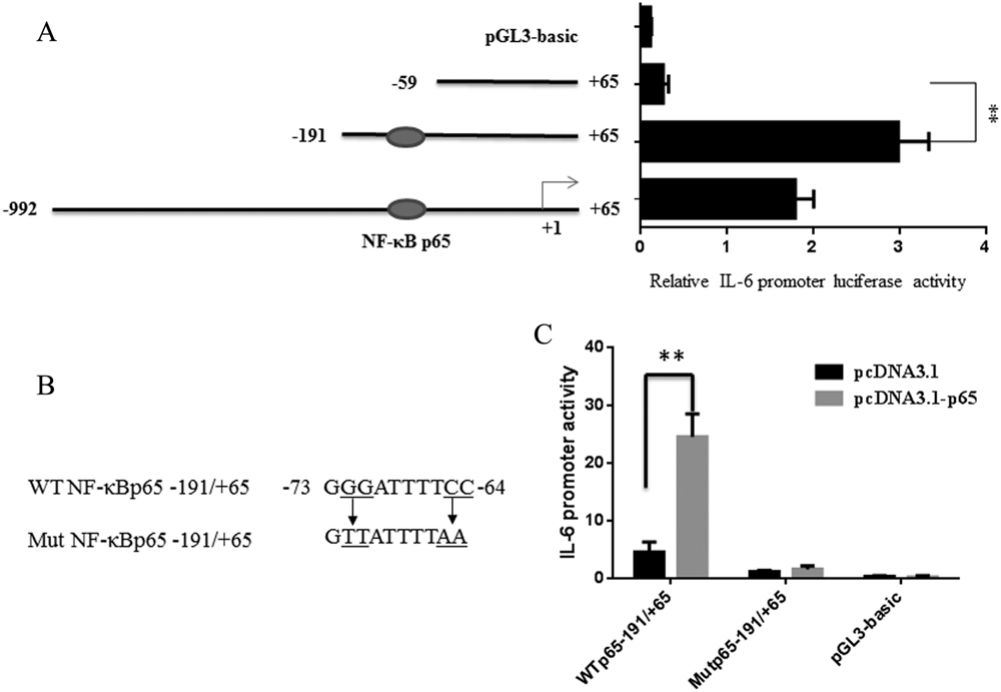
Identification of NF-κBp65 transcriptional factor controlling IL-6 gene expression. (**A**) Promoter activities of three deleted constructs of pig IL-6 gene were determined by luciferase assay in adipocytes. The nucleotides are numbered relative to the transcription start site that was assigned as +1. Schematic diagram of IL-6 promoter deletion containing the NF-κBp65 was displayed. (**B**) Mutations in the NF-κBp65 binding site in the luciferase reporter vector pGL3 (−191/+65). (**C**) Promoter activities of IL-6 (−191/+65) or a mutation at p65 (Mutp65 −191/+65) were determined by a luciferase assay after overexpression of p65. Data are expressed as the mean ± SEM of three separate experiments. Significant levels were analyzed by t- test. *P < 0.05. **P < 0.01.

**Figure 4:**
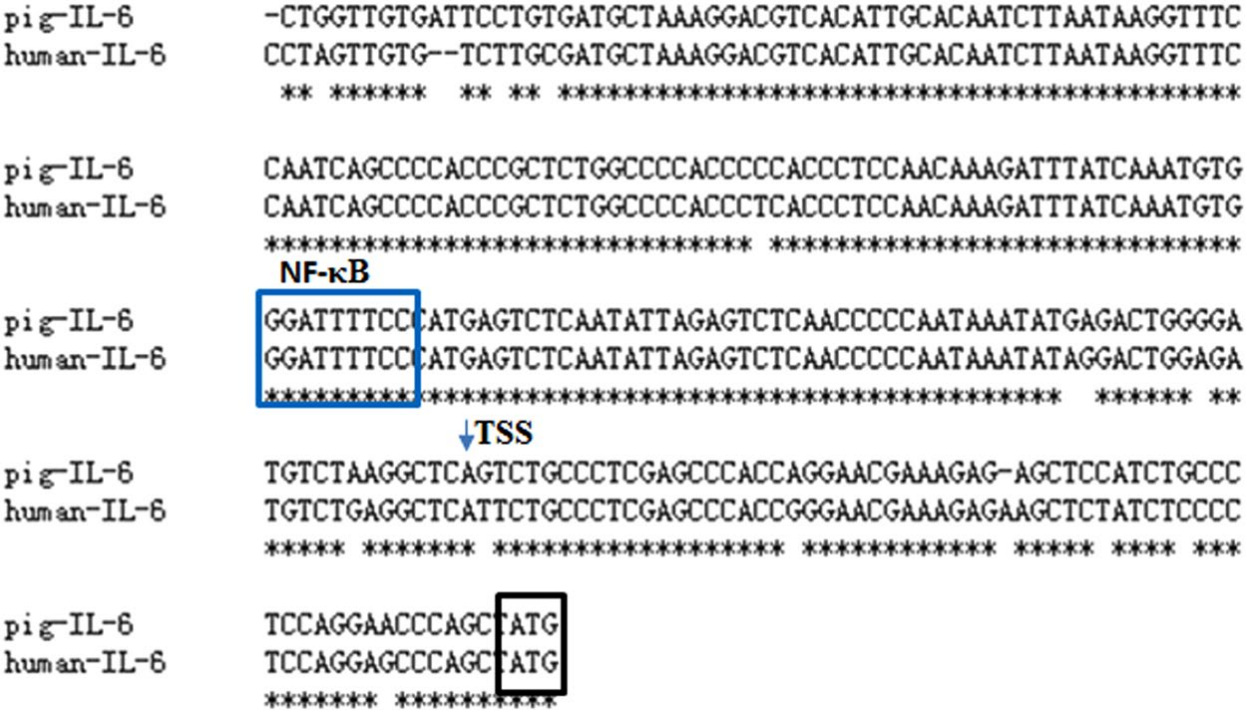
Comparison analysis between pig and human IL-6 proximal promotor regions. Arrow represents the transcriptional start site (TSS) of the pig IL-6 gene, which are identical to these of the human gene. Potential NF-κBp65 binding site was predicted and indicated with box.

To investigate the transcriptional regions of pig IL-6 gene, we cloned approximately 1.0 kb of IL-6 gene promoter region (GenBank accession number: MG786491) and generated three deletion constructs to evaluate their activity. As shown in Fig. 3A, the luciferase activities were kept in high levels in pGL3 (−191/+65) when compared with pGL3-basic and the significant differences were seen between nt −191/+65 to −59/+65 (*P* < 0.01), suggesting that the essential core region of the IL-6 promoter located at −191 bp to −59 bp. The analysis of the IL-6 gene promoter sequence in the region revealed a potential NF-κB element, which shows a high degree of conservation among pig and human (Fig. 4).

Over-expression of p65 greatly (*P* < 0.01) increased the luciferase activity of IL-6 promoter compared with the pcDNA3.1 empty vector. Then we mutated the NF-κBp65 binding site in the pGL3 (−191/+65) (Fig. 3B) and found significantly (*P* < 0.01) decreased luciferase activity of the mutated group (MutNF-κBp65 − 191/+65) compared with the wild-type group (WTNF-κBp65 − 191/+65) (Fig. 3C). In addition, the over-expression of p65 also failed to induce luciferase activity of the pGL3 (−191/+65) (Fig. 3C). These results demonstrated that pig IL-6 expression was regulated at the transcriptional level by NF-κBp65.

### GSK3β regulated IL-6 gene expression through improving NF-κB transcription activity

**Figure 5:**
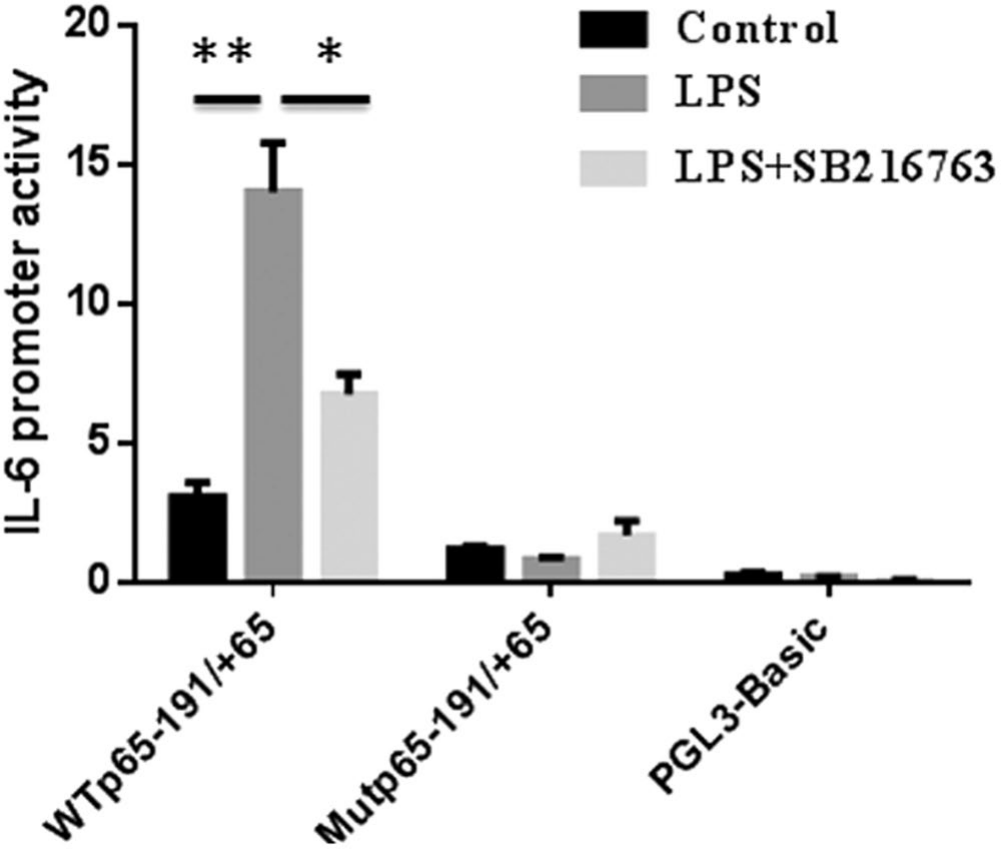
GSK3β regulated IL-6 gene expression through improving NF-κB transcription activity. Adipocytes were transfected with luciferase reporter vectors (wild-type control, WTp65 − 191/+65 or the NF-κBp65 site mutated (Mutp65 − 191/+65). At 24 h post-transfection, adipocytes were preincubated in the presence or absence of 10 μM SB216763 for 1 h and then stimulated with LPS for 24 h. After 48 h of transfection, the luciferase activity was measured using dual-luciferase reporter assay system.

To investigate the ability of GSK3β to regulate LPS-induced transcriptional activity of NF-κBp65, we examined the effect of GSK3β inhibition on the luciferase activity of the IL-6 promoter. As shown in Fig. 5, the transcription activity of the region between −191 bp and +65 bp containing NF-κBp65 element was significantly activated (*P* < 0.01) by LPS stimulation compared to negative control. In addition, use of the GSK3β inhibitor SB216763 greatly (*P* < 0.01) repressed LPS-induced luciferase activity of IL-6 promoter compared to LPS treatment group (Fig. 5).

To further determine whether GSK3β increased the transcriptional activity of IL-6 through NF-κBp65 binding sites, we analyzed the luciferase activity of IL-6 promoter with the NF-κBp65 mutation after GSK3β inhibition. No significant difference was found for the activity of the Mutp65 − 191/+65 among the LPS, LPS + SB216763 and control groups (Fig. 5), indicating that GSK3β was involved in the NF-κBp65-mediated IL-6 gene expression.

### LPS induces the binding capacity of NF-κBp65 to the IL-6 promoter

**Figure 6:**
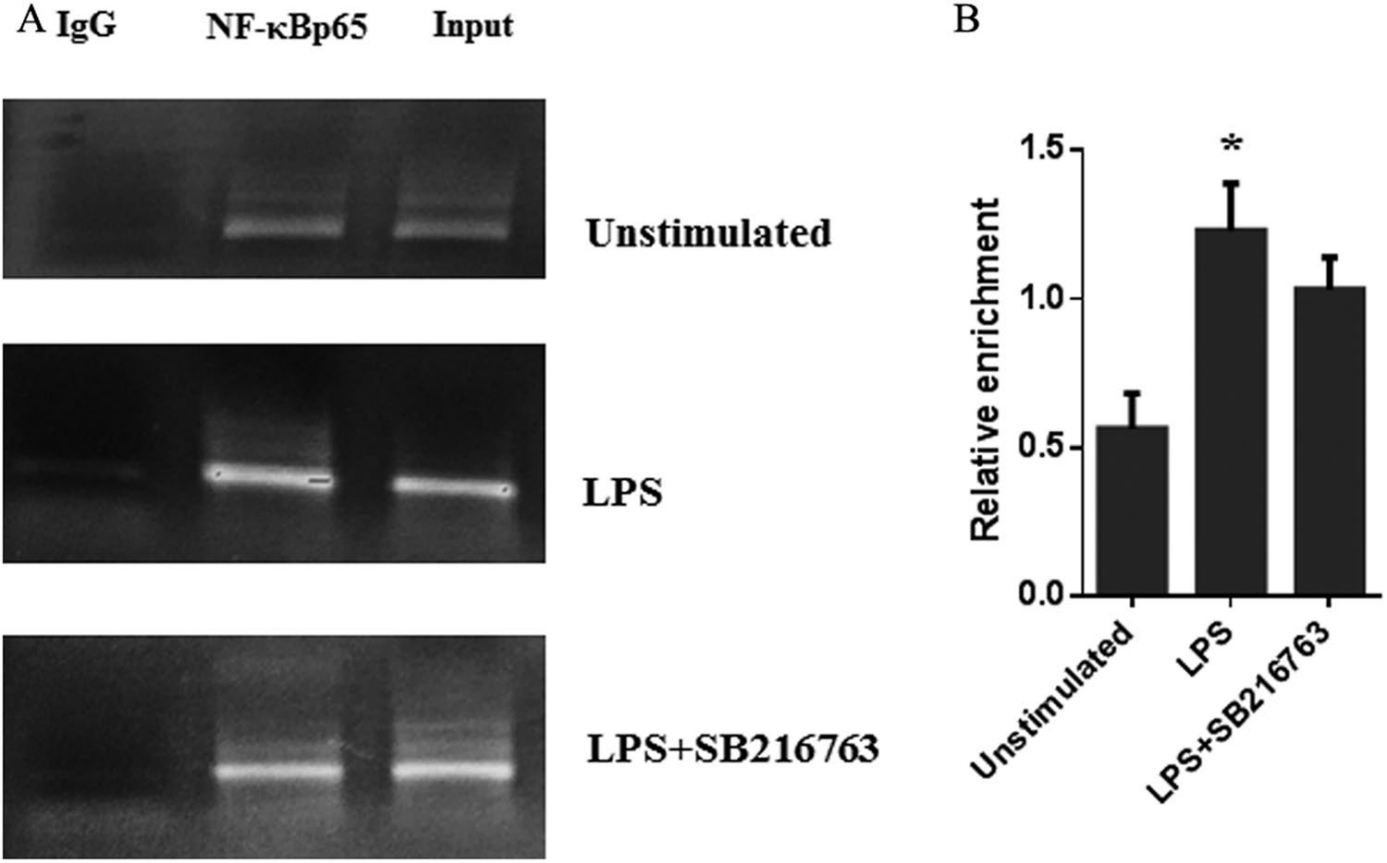
LPS affected the binding capacity of p65 to the IL-6 promoter that regulated the production of IL-6. ChIP assays were used to investigate the interaction of p65 with the IL-6 promoter in pig adipocytes. (**A**) The DNA fragments were analyzed using 2.0% Agarose gel to evaluate for qPCR specificity. (**B**) Chromatin fragments were further quantified by qPCR. Total chromatin was used as the input, and normal mouse IgG was used as the negative control. The data are presented as the percentage of PCR amplification in p65 ChIP samples and PCR amplification with input samples. Adipocytes were preincubated with medium only (Unstimulated) or preincubated in the presence or absence of 10 mM SB216763 for 60 min and then stimulated with LPS for 24 h.

To determine whether GSK3β regulated the p65 protein binding to the NF-κBp65 binding sequence, the binding capacity of NF-κBp65 with IL-6 promoter was analyzed by ChIP assay. As shown in Fig. 6A, NF-κBp65 can directly interact with the endogenous IL-6 promoter, indicating that the p65 can bind to the NF-κB site in the pig IL-6 gene promoter. Additionally, LPS significantly (*P* < 0.01) increased p65 binding to the NF-κB site confirming that LPS was involved in inducing binding to the NF-κB element (Fig. 6B). Additionally, the association of NF-κBp65 with the IL-6 promoter in LPS-stimulated adipocytes was not influenced by the GSK3β inhibition (Fig. 6B). These results suggested that NF-κBp65 involved in IL-6 transcription through directly targeting IL-6 promoter, which was mediated by LPS.

## Discussion

Previous studies have demonstrated that IL-6 is not only is a proinflammatory cytokine associated with inflammatory responses, but also a regulator on the energy and glucose metabolism. IL-6-deficient (IL-6−/−) mice displays obesity and insulin resistance compared with control mice^18^. Blocking IL-6 trans-signaling can prevent high-fat diet (HFD)-induced adipose tissue macrophage recruitment, but does not induce weight gain and improve insulin resistance^19^. Overexpression of IL-6 gene induces a significant body weight loss improved insulin sensitivity and increases the mRNA levels of lipolysis genes^20^. There is a close relationship between IL-6 and obesity-associated inflammation. However, the molecular mechanisms of IL-6 in pig have not yet fully investigated. How IL-6 regulates adipocytes biology remains poorly understood. Here, we demonstrated that the inactivation of GSK3β decreased the supernatant IL-6 productions in LPS-simulated adipocytes. We also reported that GSK3β regulated IL-6 gene expression through improving NF-κBp65 transcription activity in adipocytes.

The IL-6 proximal promoter contains functional cis-regulatory elements, such as AP-1, SP1, NF-κB and C/EBPβ, contributing to the complex regulation of the IL6 gene^21–24^. Interestingly, the transcription factors which regulated the expression of IL-6 appear to be dependent on the cell types and the signaling pathways. In innate myeloid cells, Tet2 recruites Hdac2 and mediates active repression transcription of interleukin-6 (IL-6) via histone deacetylation. And LPS stimulation increases the binding signal of Tet2 to the IL-6 promoter^25^. In C2C12 myocytes, NF-κB is necessary for IL-6 induction by LPS, and the ability of LPS to activate the IL-6 promoter is independent of the NF-IL6 and AP-1. Furthermore, LPS increases NF-κB activity by increasing the nuclear content of RelA, but not RelB^24^. In gastric epithelial cells, *H. pylori* induces IL-6 production through MAPK and NF-κB pathways^26^. However, the regulatory mechanism of IL-6 has not been studied in the pig. Our results showed that pig IL-6 expression was regulated at the transcriptional level by NF-κBp65 and p65 binding is important for pig IL-6 expression in adipocytes.

Previous studies have demonstrated that GSK3β regulates the activity of several transcription factors, including NF-κB, STAT3, CREB, and AP-1 that are important for immune function^27,28^. Inhibition of GSK3 reduces the activation of STAT3 and abolished the IL-6 production by IFN-γ administered with LPS in RAW264.7 cells^29^. In LPS-stimulated cultured primary glia, IL-6 is diminished by GSK3 inhibition through the inactivation of STAT3^30^. IFN-γ induces IL-10 production through inhibition of the activity of CREB and AP-1 which regulated by MAPKs and GSK3^5^. Methane-rich saline (MS) enhances phosphorylation of GSK-3β, which mediates the release of IL-10 and reverses the suppressed activation of NF-κB/ MAPKs in response to LPS^31^. Here we showed that LPS stimulation could induce the IL-6 production via the inhibition of GSK3β activity. GSK3β had an important role in the regulation of IL-6 production through improving NF-κB transcription activity in pig adipocytes.

GSK3β is important for the modulation of NF-κB which plays a key role in the inflammatory response and is used as an indicator of pro-inflammatory gene expression in cells exposed to bacterial infections^32,33^. Because p65 (RelA), p105 (NF-κB1) and B-cell lymphoma3-encoded protein (BCL-3) (a transcriptional co-activator of NF-κB p50 homodimer) are phosphorylated *in vitro* by GSK3β^34,35^. In mycobacteria-induced epithelial, the suppressed NF-κB action shifts the infection from a pro-inflammatory state towards an anti-inflammatory state^36^. GSK3β promotes a rapid NF-κB activation by targeting the TNFα-/p65-dependent pathway and limits the NF-κB activation in BCL-3-dependent pathways^37^. In HEK293 cells, TNFα treatment greatly increases luciferase activity of NF-κB, which is hampered by GSK-3β inhibition. And GSK-3β is critically important for NF-κB activity through modulation of NEMO phosphorylation^38^.

It has previously been demonstrated that LPS induced the phosphorylation of GSK3β through the PI(3)K-Akt-pathway in human monocytes^6^. In addition, interleukin-6 (IL-6) in turn activated Akt and inhibited GSK3β activity in airway epithelial cells^39^. In this study, we inferred that LPS lead to increase the production of IL-6, which may suppress GSK3β activity in pig adipocytes. And these results may explain why LPS increased the phosphorylation of GSK3β (Ser9). Our work revealed the important role of LPS in the regulation of IL-6 production through affecting the binding capacity of p65 to the IL-6 promoter. The association of NF-κBp65 with IL-6 promoter during LPS response was not influenced by the GSK3β inhibition. This was in agree with published studies showing that GSK3β inhibition has no effect on the DNA binding of nuclear p50 or p65 in LPS-stimulated monocytes. And GSK3β inhibition suppresses the association of NF-κBp65 to the nuclear coactivator CBP that regulates the production of IL-10 and IL-12^6^. Previous studies have been reported that Sp1 is an important bridge in binding between NF-κB and C/EBP in the IL-6 promoter^22^. In hepatocytes, JunD is able to interact with p65 and regulate NF-κB activity^40^. PI3K/PDK-1 influences the binding of JunD with p65, influencing NF-κB-dependent CCND1 gene transcription^41^. These results suggesting that GSK3β may regulate interactions between p65 and nuclear coactivators in target genes transcription.

In summary, we have identified a mechanism for the regulation of IL-6 expression by GSK3β in LPS-induced pig adipocytes. LPS increased levels of phosphorylation of GSK3β (Ser9) and then inhibited the kinase activity of GSK3β. Inhibition of GSK3β attenuated the production of IL-6 induced by LPS in pig adipocytes. Then we identified and characterized the pig IL-6 promoter, and found an NF-κBp65 element is responsible for IL-6 promoter activity. In addition, transcription activity of NF-κBp65 element was activated by LPS stimulation. Inhibition of GSK3β activation abolished the LPS-induced luciferase activity of the IL-6 promoter. LPS up-regulated the expression of IL-6 through increasing the binding capacity of p65 to the pig IL-6 promoter, and the association of NF-κBp65 with the IL-6 promoter in was not influenced by the GSK3β inhibition. Taking together, our results elucidated a critical function for GSK3β in modulating the production of IL-6 through NF-κBp65 in LPS-induced pig adipocytes.

## Materials and Methods

### Ethics statement

All research involving animals was conducted according to the Regulations for the Administration of Affairs Concerning Experimental Animals (Ministry of Science and Technology, China, revised in June 2004) and approved by the Institutional Animal Care and Use Committee at the College of Animal Science and Technology, Sichuan Agricultural University, Sichuan, China, under permit No. DKY-B20110807.

### Isolation and differentiation of porcine preadipocytes

Pig preadipocytes were isolated from the subcutaneous adipose tissues from three female Rongchang pigs (3 days old) as described before^42^. Briefly, 3 g of subcutaneous adipose tissue was washed three times with PBS, and then cut into 1-mm³ pieces and digested for 1 h (37 °C, mixed every 5 min) in 1% type I-collagen enzyme. After enzymatic digestion, cells were separated from tissue fragments by repeated centrifugation at 1000 g for 10 min, followed by filtration through a 200 µm filter and then a 50 µm Nytex filter. The cell suspension was centrifuged for 5 min at 1000 × g, and the supernatant was discarded. Then, the pellet was mixed with complete medium (10% fetal bovine serum + 2% antibiotics + DMEM) and then transferred to a culture bottle under conditions of 5% CO_2_ and 37 °C. The media were replaced after 24 h to remove impurities and dead cells.

When the preadipocytes reached full confluence, adipogenic differentiation was induced by transferring the cells into DMEM supplemented with MDI cocktail (0.5 mM 1-methyl-3-isobutylxanthine, 1 μM dexamethasone, and 10 μg/mL insulin), followed by supplementation with 10 μg/mL insulin for 1 day. The medium was replaced every two days. At day 6 of differentiation, to determine the time-course effect of LPS and SB216763, adipocytes were incubated in the presence of 1 μg/mL LPS for 0, 15, 30, 60 min or 10 μM SB216763 for 0, 30, 60 min.

### Cell transfection and luciferase assays

To examine the promoter activity of the pig IL-6 promoter, a DNA fragment of pig proximal IL-6 promoter region ranging from positions −992 to +65 bp was generated by PCR and then inserted to *Xho*I and *Hind*III restriction sites upstream of the pGL3-Basic vector (Promega). The NF-κBp65 mutated fragment was directly synthesized at Sangon Biotech (Shanghai, China) and then cloned into the pGL3-basic vector (Promega, USA) to construct the NF-κBp65 sites mutated reporter vector (MutNF-κBp65). The coding sequence of the pig p65 gene (EU399817.1) was amplified from cDNA of pig liver using primer pair (P65F, P65R, Table 1), then digested with *Xho*I and *Hind*III enzymes for sub-cloning into the pcDNA3.1(+) vector.

**Table 1:** Primer sequences used in this study.

Gene name	Primer name	Primer sequence (5′-3′)	Size(bp)	Tm (°C)
IL-6	IL6F	AAGCGCCTTCAGTCCAGT	103	60
	IL6R	GGCATCACCTTTGGCATCT		
	PIL6-992	CCGCTCGAGAAGAGGTGAGTAGTATTCTCC	1057	63
	PIL6-191	CCGCTCGAGCTGGTTGTGATTCCTGTGA	256	62
	PIL6-59	CCGCTCGAGAGTCTCAATATTAGAGTCT	124	62
	PIL6+65	CCCAAGCTTCAGACTGAGCCTTAGACAT		
	IL6-NF-KBF	CACCCTCCAACAAAGATTT	87	60
	IL6-NF-KBR	CCCAGTCTCATATTTATTGG		
p65	P65F	CCGCTCGAGATGGACGACCTCTTCCCCCT	1662	62
	P65R	CCCAAGCTTTTAGGAGCTGATCTGACTCAG		
Beta-actin	Actb-F	GGTCAAGCAGCATAATCCAAAG	158	60
	Actb-R	CAAGGGCATAGCCTACCACAA		

At day 6 of differentiation, the media of pig adipocytes in 24-well plates were changed to OPTI-MEM (Invitrogen, USA) prior to transfection. Each well was transfected with 1.0 μg of construct DNA plasmid and 50 ng of the internal control plasmid (pRL-TK, Promega) with 2 μL Lipofectamine 2000 reagent (Invitrogen, USA) according to the manufacturer’s protocol. The medium was changed at 6 h after transfection using the fresh culture medium with 10% fetal bovine serum. At 24 h post-transfection, adipocytes were preincubated with 10 mM SB216763 for 60 min and then stimulated in the presence or absence of LPS for 24 h. After 48 h of transfection, the luciferase activity was measured using dual-luciferase reporter assay system (Promega, USA).

### RNA isolation, cDNA synthesis, and qPCR Analysis

Total RNA was extracted by using Trizol reagent (Invitrogen, USA) according to the manufacturer’s protocol. The purity and concentration of the total RNA were determined with a NanoDrop (Thermo Fisher, USA) instrument. The first-strand complementary DNA (cDNA) was synthesized using 2 μg of total RNA and a PrimeScript RT Reagent Kit (Takara, Tokyo, Japan) according to the manufacturer’s protocol.

qPCR was carried out using an SYBR Green-based kit with 10 μL reaction volumes containing 5 μL of SYBR Green Real-Time PCR Master Mix (Takara, Tokyo, Japan), 0.8 μL of cDNA and 0.4 μL of each primer and using a Bio-Rad CFX96 qPCR instrument (Bio-Rad, California, USA). The qPCR procedure was as follows: initial denaturation at 95 °C for 3 min; 40 cycles at 95 °C for 30 s, alternative annealing for 20 s, and 72 °C for 15 s; and a final extension for 5 min. Melting curve analysis was used to confirm specific PCR products. An optimized comparative Ct (2^−△△Ct^) value method was used to quantify the gene expression levels relative to the expression of Beta-actin. Each RNA sample was performed in triplicate for all PCR amplification.

### Western blotting

Total proteins were extracted from adipocytes using a Cell Total Protein Extraction Kit (Sangon, Shanghai, China) and normalized with a BCA Protein Assay Kit (Sangon, Shanghai, China). 20 μg of protein samples were loaded into a 10% SDS-PAGE and then transferred from the gel to a PVDF membrane. After 1 h blockading within blocking buffer (Beyotime, Shanghai, China), membranes were incubated overnight with primary antibodies at 4 °C. After washed three times with TBST, the membranes were incubated with the secondary antibody (HRP-labeled goat anti-rabbit IgG, 1:2000, Santa Cruz, USA) for 2 h at 37 °C. The results were visualized using an ECL detection system (BeyoECL Plus, Beyotime, Shanghai, China). Primary antibodies contain anti-Ser9-GSK3β (1:400, Santa Cruz, USA), anti-GSK3β (1:1000, Cell Signaling Technology, USA) and anti-Ser641-GS (1:1000, Cell Signaling Technology, USA) and β-actin (1:1000, Santa Cruz, USA).

### Enzyme-linked immunosorbent assay (ELISA)

The cell supernatant was centrifuged at 3000 rpm for 10 min and stored at −80 °C until analysis. Then the culture medium concentrations of IL-6 were measured using porcine IL-6 ELISA kit (Gersion Bio-Technology Co. Ltd, Beijing, China) according to the manufacturer’s instructions on a microplate reader (Thermo, America). Each supernatant sample was performed in triplicate for all Enzyme-linked immunosorbent assays.

### Chromatin immunoprecipitation (ChIP) assay

Chromatin immunoprecipitation (ChIP) assay was performed on adipocytes using the ChIP Assay Kit (Beyotime, Jiangsu, China) as described previously^15^. Briefly, porcine preadipocytes were seeded in 6-well plates and differentiated for 6 days, then the adipocytes were fixed in 1% formaldehyde at 37 °C for 20 min and neutralized with glycine for 5 min. After washing with cold PBS, the cells were scraped and collected. And then nuclear lysates were sonicated on ice to break the genome into 200–1000 bp. Chromatin complexes were immunoprecipitated overnight at 4 °C with the NF-κBp65 antibody (Cell signaling technology, USA), and then pulled-down using the Protein A+G Agarose beads (Beyotime, China), washed and then eluted. The DNA was extracted using phenol/chloroform method. After purification, the 2 μL DNA obtained from the immunoprecipitation was amplified using primers flanking the NF-κB binding sites (IL6-NF-KBF, IL6-NF-KBR, Table 1). The DNA fragments were analyzed using 2.0% Agarose gel to evaluate for qPCR specificity and further quantified with ChIP-qPCR. Signals were normalized using 1% input.

## Electronic supplementary material

Figure S1
