# Supplementary material for: GSK3β inhibition attenuates LPS-induced IL-6 expression in porcine adipocytes
Source: Sci Rep. 2018 Oct 29;8:15967. doi: 10.1038/s41598-018-34186-0 (PMC6206029; doi:10.1038/s41598-018-34186-0)

GSK3β inhibition attenuates LPS-induced IL-6 expression in porcine adipocytes

By

Linjie Wang, Xueying Li, Yan Wang*

From the College of Animal Science and Technology

Sichuan Agricultural University

Figure S1: Whole membrane images for Figures 1A.


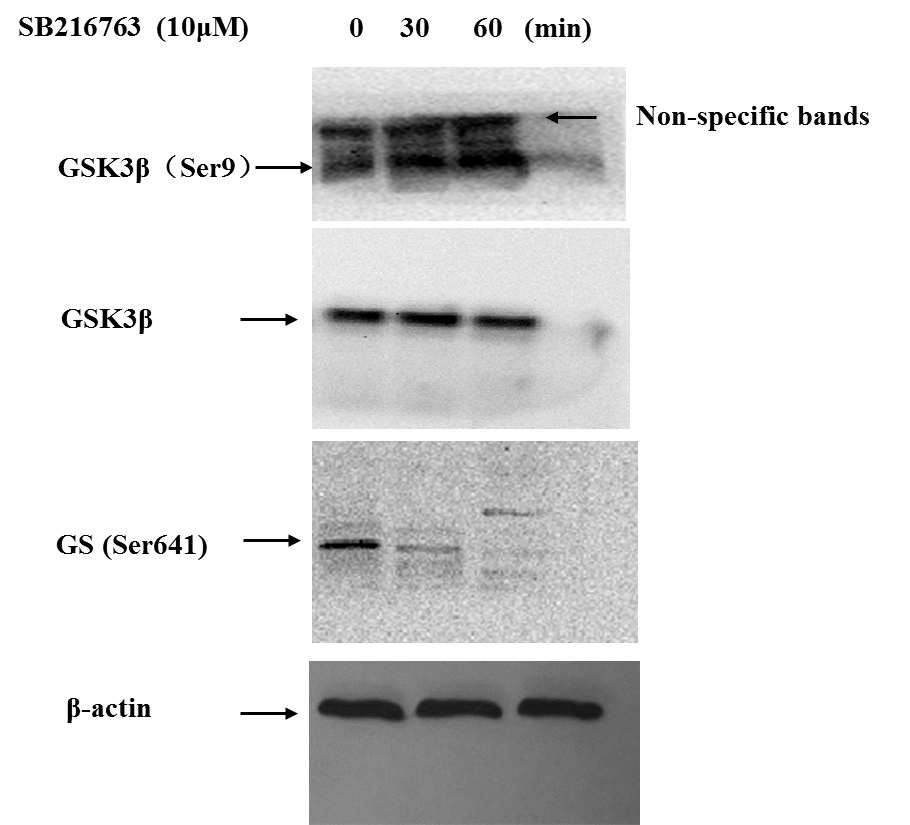


Whole membrane images for Figures 1D.


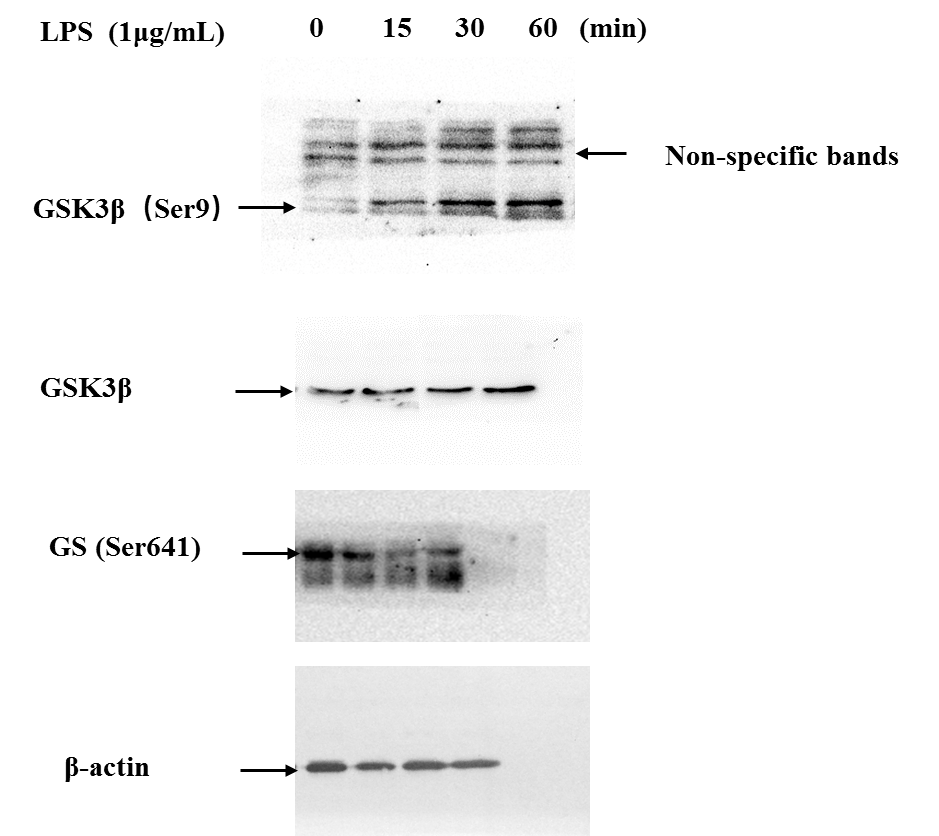

Supplement: Supplementary file 1 — Figure S1 [file 41598_2018_34186_MOESM1_ESM.docx]
